# Application of supervised machine learning algorithms to predict the risk of hidden blood loss during the perioperative period in thoracolumbar burst fracture patients complicated with neurological compromise

**DOI:** 10.3389/fpubh.2022.969919

**Published:** 2022-09-26

**Authors:** Bo Yang, Lin Gao, Xingang Wang, Jianmin Wei, Bin Xia, Xiangwei Liu, Peng Zheng

**Affiliations:** ^1^Department of Orthopedics, Baoji City Hospital of Traditional Chinese Medicine, Baoji, China; ^2^Department of Spine, School of Medicine, The Honghui-Hospital, Xi'an Jiaotong University, Xi'an, China; ^3^Department of Cardiology, The First Affiliated Hospital of Nanjing Medical University, Nanjing, China

**Keywords:** application, machine learning, hidden blood loss, risk factors, thoracolumbar burst fracture

## Abstract

**Background:**

Machine learning (ML) is a type of artificial intelligence (AI) and has been utilized in clinical research and practice to construct high-performing prediction models. Hidden blood loss (HBL) is prevalent during the perioperative period of spinal treatment and might result in a poor prognosis. The aim of this study was to develop a ML-based model for identifying perioperative HBL-related risk factors in patients with thoracolumbar burst fracture (TBF).

**Methods:**

In this study, single-central TBF patients were chosen. The medical information on patients, including clinical characteristics, laboratory indicators, and surgery-related parameters, was extracted. After comparing various ML model algorithms, we selected the best model with high performance. The model was validated using the internal validation set before performing recursive feature elimination (RFE) to determine the importance of HBL-related risk factors. The area under the receiver operating characteristic (AUC) curve, accuracy (ACC), sensitivity, and specificity were reported as critical model measures for evaluating predictive performance.

**Results:**

In this study, 62 (38.5%) of the 161 TBF patients were positive for HBL. There was a significant statistical difference in age, body mass index (BMI), diabetes, hypertension, Beta (percentage of vertebral restoration), duration of operation, and other pre-operative laboratory indicators between the HBL-positive and HBL-negative groups. Nine ML-based models were built and validated, with the Random Forest model having the greatest AUC in both the training set (0.905) and internal validation set (0.864). Furthermore, following RFE, age, duration of operation, Beta, pre-operative fibrinogen (Fib), and activated partial thromboplastin time (APTT) were identified as the five main important risk factors in patients with TBF during the perioperative period.

**Conclusion:**

In this study, we built and validated ML algorithms for an individualized prediction of HBL-related risk factors in the perioperative period of TBF. The importance of HBL-related risk factors could be determined, which contributes to clinicians' decision-making and improves perioperative management.

## Introduction

A spinal compression fracture is a type of severe spinal injury that falls under the category of traumatic spine fractures. Thoracolumbar burst fracture (TBF) accounts for roughly 25–48% of all thoracolumbar spinal fractures, as well as the highest proportion of traumatic spine fractures (1, 2). TBF commonly has a detrimental impact on patients, such as a shorter lifespan and a worse quality of life (1–3). As a result, spinal surgery is typically performed to rebuild spinal stability, relieve spinal cord compression, and restore spinal function, as well as to improve prognosis and prevent relevant complications (4). Although there is still no consensus on the need for surgical treatment for the small percentage of patients with TBF that are without neurological compromise, the majority of TBF patients have varying degrees of neurological compromise. In clinical practice, surgical interventions for TBF are usually indicated if injuries complicated with neurological compromise.

It is particularly important to note that blood loss during the intraoperative period has become a prevalent clinical concern in TBF patients (5, 6). A reduction in hemoglobin (Hb) content observed in fracture patients cannot be entirely explained by dominant blood loss during the perioperative period (6). As a result, the concept of hidden blood loss (HBL) during the perioperative period was originally presented in 2000 (7) and is progressively gaining acceptance among academics. According to this theory, HBL and dominant blood loss work together to explain why Hb levels are much lower. Numerous studies (6–8) have found a link between HBL and total blood loss (TBL) in patients undergoing joint and spine surgery. According to the findings, HBL accounted for around half of TBL. Consequently, identifying and controlling HBL risk factors during the perioperative period is critical for improving patients' prognosis and clinical administration efficacy. Although few studies have identified HBL-related risk factors under TBF (5, 6, 9), there are limited publications on determining the importance rank of HBL-related risk factors.

Machine learning (ML) belongs to a kind of artificial intelligence (AI) that is a new multidisciplinary technology based on computer science that can automatically learn and continuously improve depending on the identification of patterns and complex relationship (9–11). Its goal is fast decision-making with minimum intervention from humankind. Because of their superior prediction performance over traditional statistical tools, ML algorithms are increasingly being used in the medical field for some clinical determinations. Unfortunately, there have been few studies that use the ML algorithm to train the model and predict the high-risk factors for HBL in TBF patients. Therefore, in this study, ML-based models were constructed and validated for the importance identification of HBL-related risk factors in patients with single-level TBF.

## Methods

### Patient population, inclusion, and exclusion criteria

This study protocol was authorized and supervised by the Research Ethics Review Board of the Baoji City Hospital of Traditional Chinese Medicine (Baoji, China). The Approval Number was No. 2020YTH8H2. Patients (from March 2013 to March 2019) who were diagnosed with symptomatic single-level TBF in clinic were chosen. In this study, the specified inclusion criteria are involved in the following aspects: (1) patients over the age of eighteen; (2) Denis classification of TBF (T11–L2): type B with vertebral compression more than 50%, local kyphosis angle >30° (12); (3) patients' Thoracolumbar Injury Classification and Severity Score (TLICS) more than 5 (13, 14); and (4) Gaines load score < 6 (15). The specified exclusion criteria are involved in the following aspects: (1) patients with internal organ diseases (such as liver or kidney dysfunction); (2) patients with abnormal coagulation function; (3) patients with spinal surgical history; (4) patients with spontaneous cerebrospinal fluid leakage; and (5) patients who used hemostatic medications during the perioperative period. We categorized HBL lower than 470 mL as the HBL-negative group, and otherwise, as the HBL-positive group, as described previously (16).

### Patients' data collection

The medical information of TBF surgery patients was collected in this study, which included essential information [age, gender, body mass index (BMI), smoking, and drinking], chronic diseases [hypertension, diabetes, and chronic obstructive pulmonary disease (COPD)], and history of blood transfusion and chronic steroid. Moreover, pre-operative scoring indications, including the visual analog score (VAS), Japanese Orthopedic Association (JOA) score, and 12-Item Short Form Health Survey (SF-12), were obtained. Pre-operative laboratory indicators, including hematocrit (Hct), Hb, albumin (ALB), fibrinogen (Fib), activated partial thromboplastin time (APTT), serum potassium, serum calcium, and serum sodium, were obtained. Meanwhile, we obtained surgery-related and intraoperative indicators from the electronic medical record (EMR) of the hospital. The primary indicators were operation time, levels of fused vertebrae, total time from admission to operation, and intraoperative fluid management strategy. The information on post-operative complications and scoring indications was collected, such as cerebrospinal fluid (CSF) leak, deep venous thrombosis (DVT), urinary tract infection, superficial infection, delayed wound healing, and failure of pedicle screw internal fixation, as well as VAS, SF-12, and JOA scoring.

Subsequently, we calculated percentages of vertebral restoration (Beta) to observe if fractured vertebral height had an impact on HBL. Based on X-ray parameters of patients during the pre-operative and post-operative periods, the value of Beta could be estimated using the following formula:


$$\begin{array}{l} Beta=\left(a_{4}-a_{1}\right)/a_{5}\times 100\%,a_{5}=\left(a_{2}+a_{3}\right)/2 \end{array}$$

**Figure 1:**
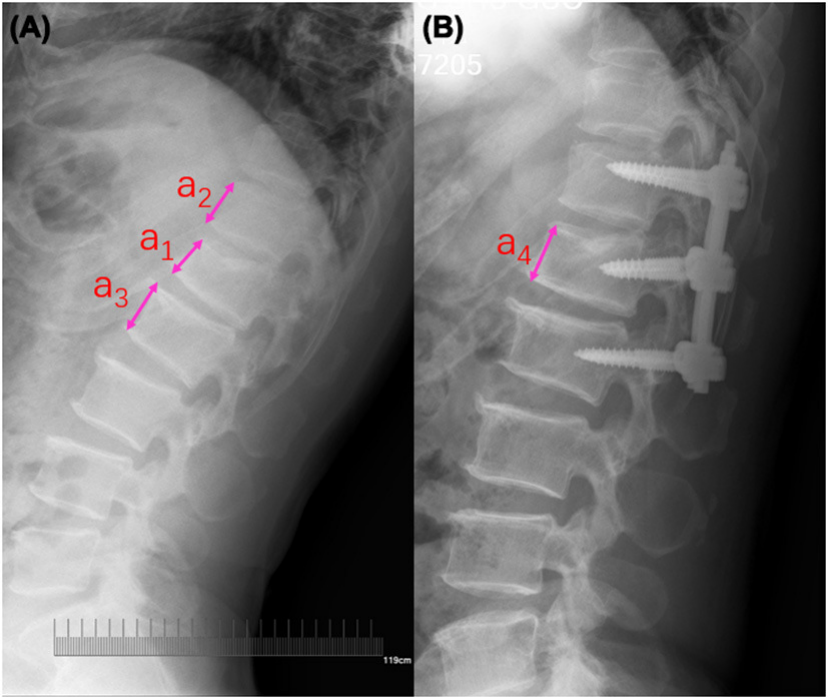
Radiographic measurement of the fractured vertebral body. **(A)** Pre-operative lateral radiograph; a_1_ is defined as the height of the fractured vertebra; the heights of the upper and lower anterior vertebrae that were adjacent to the fractured vertebra were presented as a_2_ and a_3_, respectively. **(B)** Post-operative lateral radiograph; a_4_ is defined as the height of the post-operative vertebra; the average height of a_2_ and a_3_ is calculated and defined as the predicted height of fractured vertebra (a_5_).

Here, a_1_ is defined as the height of the fractured vertebra; the heights of the upper and lower anterior vertebrae that were adjacent to the fractured vertebra were presented as a_2_ and a_3_, respectively; a_4_ is defined as the height of the post-operative vertebra; the average height of a_2_ and a_3_ is calculated and defined as the predicted height of the fractured vertebra (a_5_). Figure 1 depicts the detected method for these parameters.

### HBL evaluation

According to a previous study (17), we first calculated patients' blood volume (PBV) by the following method:


$$\begin{array}{l} PBV=k1\times height\left(m\right)+k2\times weight\left(kg\right)+k3 \end{array}$$

Here, the relevant coefficients are displayed. k1 = 0.3669, k2 = 0.03219, and k3 = 0.6041 (for male); k1 = 0.3561, k2 = 0.03308, and k3 = 0.1833 (for female).

According to the method proposed by Gross previously (18), we calculated TBL by the following method:


$$\begin{array}{l} TBL=PBV\times \left(Hct_{\text{pre}}-Hct_{\text{post}}\right)/Hct_{\text{ave}} \end{array}$$

Here, the Hct_pre_ and Hct_post_ were the Hct on pre-operative day 1 and post-operative day 3, respectively. Then, Hct_ave_ was calculated by averaging the values of Hct_pre_ and Hct_post_.

Subsequently, we calculated patients' visible blood loss (VBL) during the perioperative period by the following method:


$$\begin{array}{lll} VBL\; & = & \;intraoperative\;blood\;loss\;\left(IBL\right)\;+\;postoperative\;drainage;\\ IBL\; & = & \;suction\;containers\;+soaked\;gauzes\;+\;soaked\;sponges \end{array}$$

Consequently, we calculated patients' HBL during the perioperative period by the first following method. When patients received blood transfusions during the perioperative period, HBL was calculated by the second following method.


$$\begin{array}{l} \text{The first method is}:\text{ HBL }=\text{ TBL }-\text{ VBL}\\ \text{The second method is}:\text{ HBL }=\text{ TBL }-\text{ VBL}\\ +\text{ autologous blood transfusion}\\ +\text{ allogeneic blood transfusion} \end{array}$$

### Model development and validation

**Figure 2:**
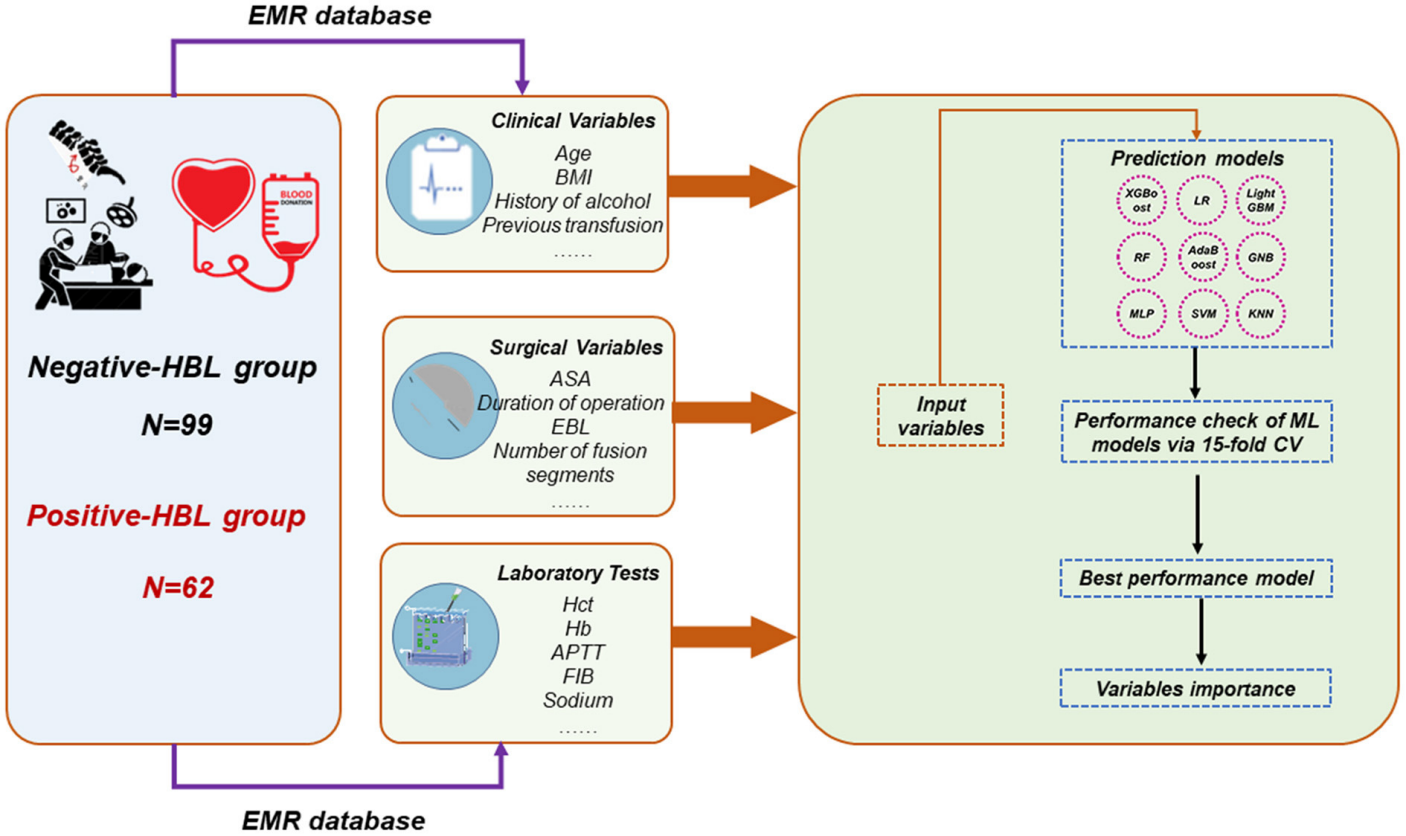
Overall flowchart. The final prediction model was determined by the maximum AUC and ACC. HBL, hidden blood loss; EMR, electronic medical record; AUC, area under the receiver operating characteristic curve; ACC, accuracy; RF, random forest; GNB, Gaussian NB; MLP, multi-layer perceptron neural network; SVM, support vector machine; KNN, k-nearest neighbors; LR, logistic regression.

ML algorithm-based models were built to predict HBL-related risk factors in patients with TBF during the perioperative period. The data collected from the single center was randomly divided into two cohorts (including the training set and internal validation set). In order to avoid overfitting and find the optimal hyperparameters, 15-fold cross-validation (CV) was adopted on the training set. In this study, ML-based models, including XGBoost, logistic regression, LightGBM, Random Forest (RF), support vector machine (SVM), AdaBoost, Gaussian NB (GNB), k-nearest neighbors (KNN), and multi-layer perceptron neural network (MLP), were developed for predicting HBL-related risk factors. The model with the highest prediction performance was determined as the final prediction model. In this process, we performed the Shapley Additive explanations (SHAP) values-based recursive feature elimination (RFE) algorithm to obtain crucial features. This will make the developed model feasible for application. The ML-based classifiers are implemented using the python library Sklearn69 package (19, 20). The evaluation-related indicators for the model performance, involving the area under the receiver operating characteristic curve (AUC) value, sensitivity, specificity, accuracy, and F-1 score, were reported. Figure 2 shows the flowchart of this study.

### Statistical analysis

The continuous variables being normally distribution were exhibited as means ± standard deviations (SD), and otherwise, as median (interquartile spacing). The student's *t*-test and the Mann-Whitney U-test were carried out for the two group comparisons of continuous variables. The categorical variables were exhibited as numerical values and proportions and compared using χ^2^-test or Fisher's exact test. In the present study, SPSS software (22.0 version) was used to carry out all statistical analyses. The values of *p* < 0.05 indicated a significant statistical difference.

## Results

### Patients' baseline characteristics

After the selection based on the set inclusion and exclusion criteria, a total of 161 patients diagnosed with TBF and treated with spinal surgery were eventually included in this study. Among these individuals, 62 (38.51%) cases were in the HBL-positive group and 99 cases were in the HBL-negative group. There were 113 (70.2%) females and 48 (29.8%) males among the 161 patients, with an average age of 41.3 ± 10.6.

The results of statistical analysis showed that there were remarked differences in age, BMI, current smoking, hypertension, diabetes, Beta, duration of operation, total time from admission to surgery, pre-operative Hct, pre-operative Hb, pre-operative APTT, and pre-operative Fib between the HBL-positive and HBL-negative groups (Table 1). There was no significant difference in overall post-operative complications between the two groups, except superficial infection (Table 2). Furthermore, there were no differences between the two groups in terms of the indicators of scoring (VAS score, JOA score, and SF-12) in the pre-operative and post-operative period, levels of fusion, and intraoperative fluid management strategy (Table 1; Figure S1).

**Table 1:** Comparison of variables between the two groups.

	**Total**	**Positive-HBL group (n = 62)**	**Negative-HBL group (n = 99)**	***P*-value**
Number of patients	161	62	99	
Age (year)	41.3 (10.6)	48.9 (8.6)	36.6 (8.8)	< 0.001
**Sex (%)**				
Female	113 (70.2)	42 (67.7)	71 (71.7)	0.592
Male	48 (29.8)	20 (32.3)	28 (28.3)	
BMI (kg/m^2^)	24.8 (22.9, 28.0)	26.1 (23.1, 29.4)	24.4 (22.8, 27.2)	0.042
**Current smoking (%)**				
No	80 (49.7)	17 (27.4)	63 (63.6)	< 0.001
Yes	81 (50.3)	45 (72.6)	36 (36.4)	
**History of alcohol (%)**				
No	72 (44.7)	29 (46.8)	43 (43.4)	0.678
Yes	89 (55.3)	33 (53.2)	56 (56.6)	
**Hypertension (%)**				
No	62 (38.5)	13 (21.0)	49 (49.5)	< 0.001
Yes	99 (61.5)	49 (79.0)	50 (50.5)	
**Diabetes (%)**				
No	76 (47.2)	20 (32.3)	56 (56.6)	0.003
Yes	85 (52.8)	42 (67.7)	43 (43.4)	
**Chronic steroid use (%)**				
No	137 (85.1)	54 (87.1)	83 (83.8)	0.572
Yes	24 (14.9)	8 (12.9)	16 (16.2)	
**COPD (%)**				
No	157 (97.5)	61 (98.4)	96 (97.0)	0.574
Yes	4 (2.5)	1 (1.6)	3 (3.0)	
**Previous transfusion (%)**				
No	155 (96.2)	59 (95.2)	96 (97.0)	0.556
Yes	6 (3.8)	3 (4.8)	3 (3.0)	
Time from admission to surgery (d)	1.0 (0.0, 1.0)	1.0 (0.0, 1.0)	0.0 (0.0, 1.0)	< 0.001
Duration of operation (min)	170.1 (18.1)	181.6 (13.5)	162.9 (16.8)	< 0.001
Beta	30.0 (27.8, 33.0)	31.1 (28.0, 35.1)	28.9 (27.6, 30.3)	< 0.001
Levels of fusion	1.0 (0.0, 1.0)	1.0 (0.0, 1.0)	0.0 (0.0, 1.0)	0.089
Intraoperative infusion of crystalloids (mL)	1,634.7 (418.1)	1,681.7 (424.4)	1,605.2 (411.4)	0.261
Intraoperative infusion of colloids (mL)	802.8 (310.1)	816.5 (317.6)	794.3 (305.0)	0.66
**Autologous blood transfusion (%)**				
No	128 (79.5)	48 (77.4)	80 (80.8)	0.604
Yes	33 (20.5)	14 (22.6)	19 (19.2)	
**Allogeneic blood transfusion (%)**				
No	128 (79.5)	50 (80.6)	78 (78.8)	0.776
Yes	33 (20.5)	12 (19.4)	21 (21.2)	
Pre-operative Hct (%)	40.8 (3.9)	41.6 (4.2)	40.3 (3.6)	0.034
Pre-operative Hb (g/L)	122.9 (15.4)	119.9 (13.1)	124.9 (16.4)	0.046
Pre-operative ALB (g/L)	39.7 (4.5)	40.0 (5.0)	39.5 (4.1)	0.459
Pre-operative APTT (s)	34.3 (3.0)	33.2 (3.2)	35.0 (2.6)	< 0.001
Pre-operative Fib (mg/dL)	4.6 (0.9)	4.3 (0.8)	4.8 (0.9)	< 0.001
Pre-operative serum sodium (mmol/L)	136.0 (9.0)	136.7 (10.5)	135.5 (7.8)	0.438
Pre-operative serum potassium (mmol/L)	3.8 (0.2)	3.8 (0.2)	3.8 (0.2)	0.961
Pre-operative serum calcium (mmol/L)	2.1 (0.3)	2.1 (0.1)	2.1 (0.4)	0.395

BMI, body mass index; COPD, chronic obstructive pulmonary disease; Beta. percentage of vertebral restoration; Hct, hematocrit; Hb, hemoglobin; ALB, albumin; APTT, activated partial thromboplastin time; Fib, fibrinogen.

**Table 2:** Comparison of complications between the positive-HBL group and negative-HBL group.

**Parameters**	**Positive-HBL group (*n* = 62)**	**Negative-HBL group (*n* = 99)**	***P-*value**
**Complications (%)**			
CSF leak	3 (4.84%)	5 (5.05%)	0.892
DVT	4 (6.45%)	7 (7.07%)	0.294
Urinary tract infection	1 (1.61%)	0 (0.00%)	0.495
Superficial infection	4 (6.45%)	1 (1.01%)	0.023^*^
Delayed wound healing	2 (3.23%)	2 (2.02%)	0.729
Failure of pedicle screw internal fixation	0 (0.00%)	1 (1.01%)	0.234
Total	11 (22.58%)	16 (16.16%)	0.087

CSF, cerebrospinal fluid; DVT, deep venous thrombosis.

* P < 0.05.

### Predictive performance of machine learning algorithm

**Figure 3:**
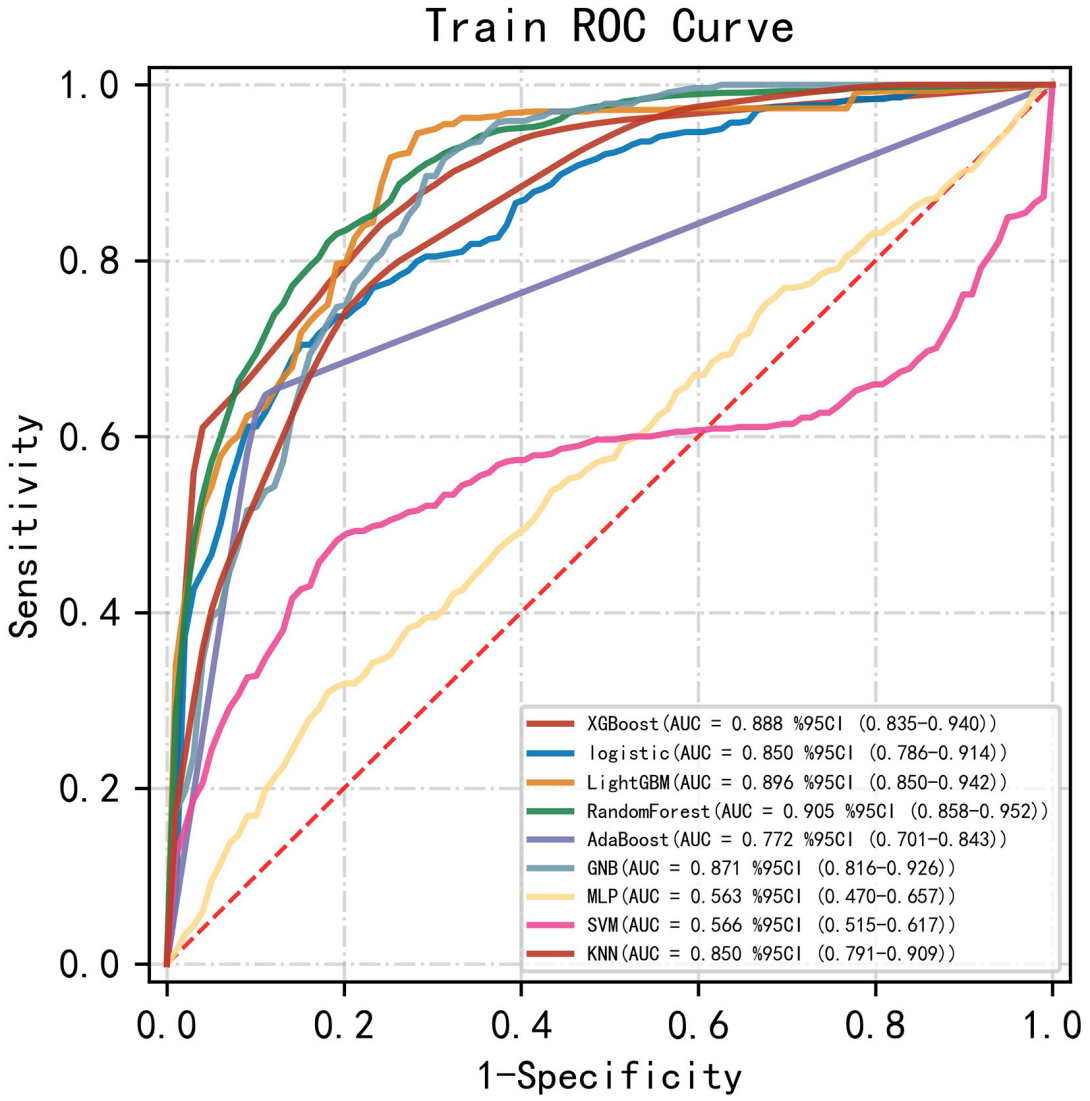
Comparisons of different machine learning models in the area under the receiver operating characteristic curve (AUC) in the training set. RF, random forest; GNB, Gaussian NB; MLP, multi-layer perceptron neural network; SVM, support vector machine; KNN, k-nearest neighbors.

**Figure 4:**
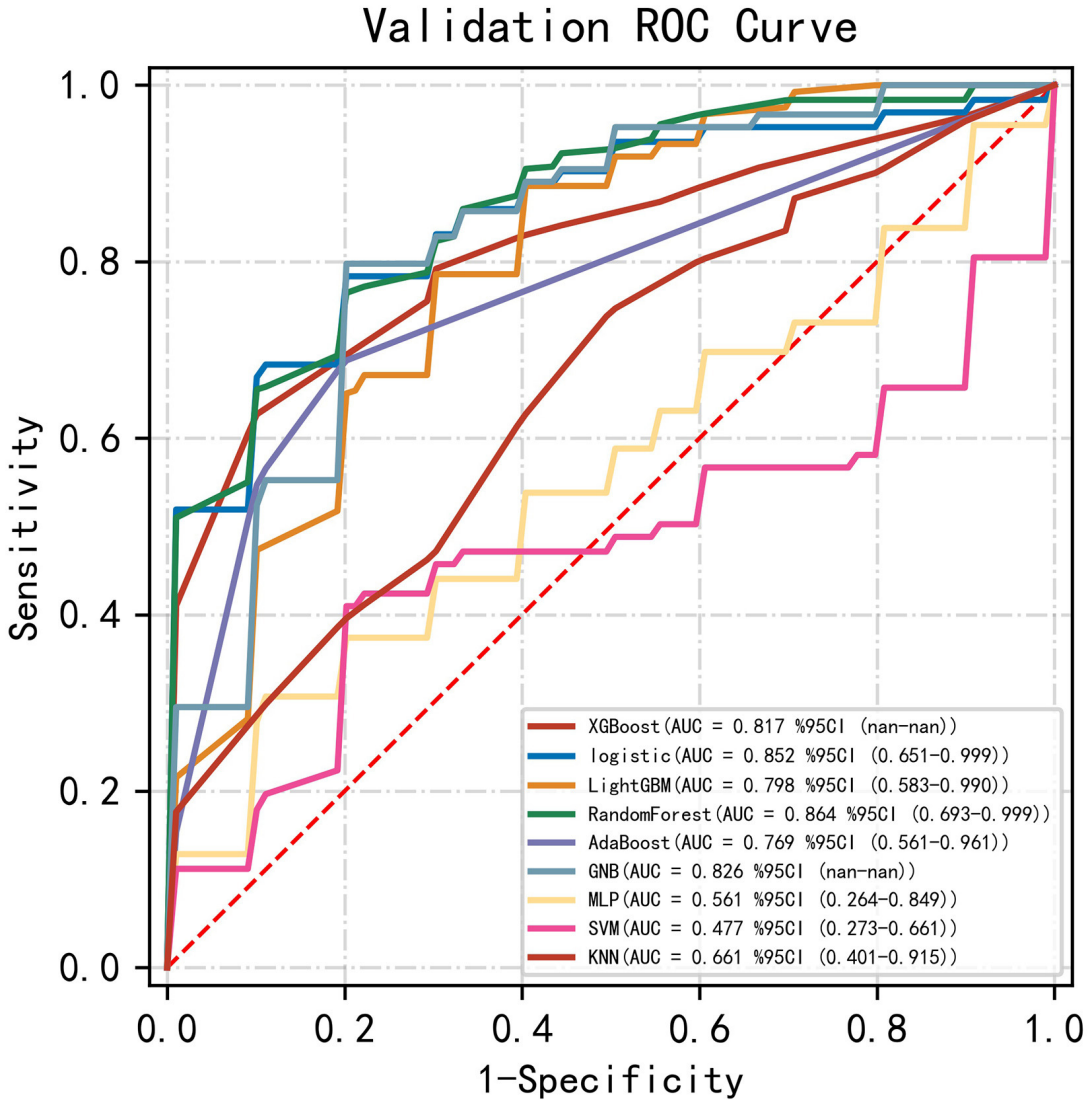
Comparisons of different machine learning models in the area under the receiver operating characteristic curve (AUC) in the validating set. RF, random forest; GNB, Gaussian NB; MLP, multi-layer perceptron neural network; SVM, support vector machine; KNN, k-nearest neighbors.

Among the nine ML-based models we developed and validated in this study, the RF model algorithm for the initial prediction of HBL exhibited excellent performance, with the highest value of AUC (0.905) (Figure 3). Furthermore, the RF model's sensitivity, specificity, and accuracy in the training set were 0.839, 0.828, and 0.827, respectively (Table 3). When the models developed in the training set were performed for internal validation, the RF model still displayed the best performance (the highest AUC = 0.864), with the highest accuracy of 0.783 (Figure 4; Table 4). Accordingly, the RF algorithm was used as the ultimate model for predicting the risk of HBL in patients with TBF.

**Table 3:** Model parameters in training set.

	**AUC**	**Sensitivity**	**Specificity**	**Accuracy**	**PPV**	**NPV**	**F1-Score**
XGBoost	0.888 (0.01)	0.830 (0.11)	0.781 (0.11)	0.759 (0.07)	NA	0.811 (0.11)	NA
Logistic	0.850 (0.03)	0.749 (0.02)	0.834 (0.04)	0.794 (0.02)	0.737 (0.05)	0.832 (0.01)	0.742 (0.03)
LightGBM	0.896 (0.08)	0.925 (0.09)	0.846 (0.08)	0.868 (0.05)	0.796 (0.08)	0.935 (0.05)	0.851 (0.07)
RF	0.905 (0.02)	0.839 (0.10)	0.828 (0.09)	0.827 (0.02)	0.777 (0.07)	0.874 (0.04)	0.799 (0.03)
AdaBoost	0.772 (0.01)	0.645 (0.02)	0.899 (0.01)	0.615 (0.00)	NA	0.615 (0.00)	NA
GNB	0.871 (0.02)	0.941 (0.01)	0.699 (0.04)	0.785 (0.02)	0.659 (0.03)	0.935 (0.01)	0.775 (0.02)
MLP	0.563 (0.12)	0.495 (0.22)	0.695 (0.16)	0.611 (0.03)	NA	0.693 (0.05)	NA
SVM	0.566 (0.38)	0.527 (0.43)	0.859 (0.13)	0.727 (0.09)	NA	0.787 (0.14)	NA
KNN	0.850 (0.02)	0.771 (0.03)	0.788 (0.03)	0.744 (0.02)	0.832 (0.03)	0.723 (0.01)	0.800 (0.03)

Data are shown as means ± standard deviations (SD).

AUC, area under the receiver operating characteristic curve; PPV, positive prediction value; NPV, negative prediction value; RF, random forest; GNB, Gaussian NB; MLP, multi-layer perceptron neural network; SVM, support vector machine; KNN, k-nearest neighbors; NA, Not applicable.

**Table 4:** Model parameters in validating set.

	**AUC**	**Sensitivity**	**Specificity**	**Accuracy**	**PPV**	**NPV**	**F1-Score**
XGBoost	0.817 (0.11)	0.779 (0.20)	0.850 (0.15)	0.727 (0.09)	NA	0.778 (0.11)	NA
Logistic	0.852 (0.08)	0.902 (0.11)	0.816 (0.14)	0.765 (0.08)	0.685 (0.09)	0.826 (0.10)	0.776 (0.08)
LightGBM	0.798 (0.10)	0.852 (0.13)	0.758 (0.11)	0.758 (0.10)	0.670 (0.12)	0.855 (0.09)	0.740 (0.10)
RF	0.864 (0.09)	0.867 (0.16)	0.814 (0.16)	0.783 (0.09)	0.741 (0.15)	0.844 (0.10)	0.793 (0.14)
AdaBoost	0.769 (0.09)	0.650 (0.19)	0.889 (0.07)	0.615 (0.02)	NA	0.615 (0.02)	NA
GNB	0.826 (0.10)	0.905 (0.10)	0.776 (0.14)	0.745 (0.14)	0.652 (0.16)	0.897 (0.12)	0.750 (0.13)
MLP	0.561 (0.16)	0.557 (0.27)	0.749 (0.25)	0.553 (0.12)	NA	0.635 (0.15)	NA
SVM	0.477 (0.34)	0.538 (0.44)	0.827 (0.18)	0.659 (0.12)	NA	0.730 (0.12)	NA
KNN	0.661 (0.14)	0.640 (0.32)	0.738 (0.21)	0.640 (0.10)	0.595 (0.33)	0.652 (0.07)	0.535 (0.30)

Data are shown as means ± standard deviations (SD).

AUC, area under the receiver operating characteristic curve; PPV, positive prediction value; NPV, negative prediction value; RF, random forest; GNB, GaussianNB; MLP, multi-layer perceptron neural network; SVM, support vector machine; KNN, k-nearest neighbors; NA, Not applicable.

### Relative importance of variables

**Figure 5:**
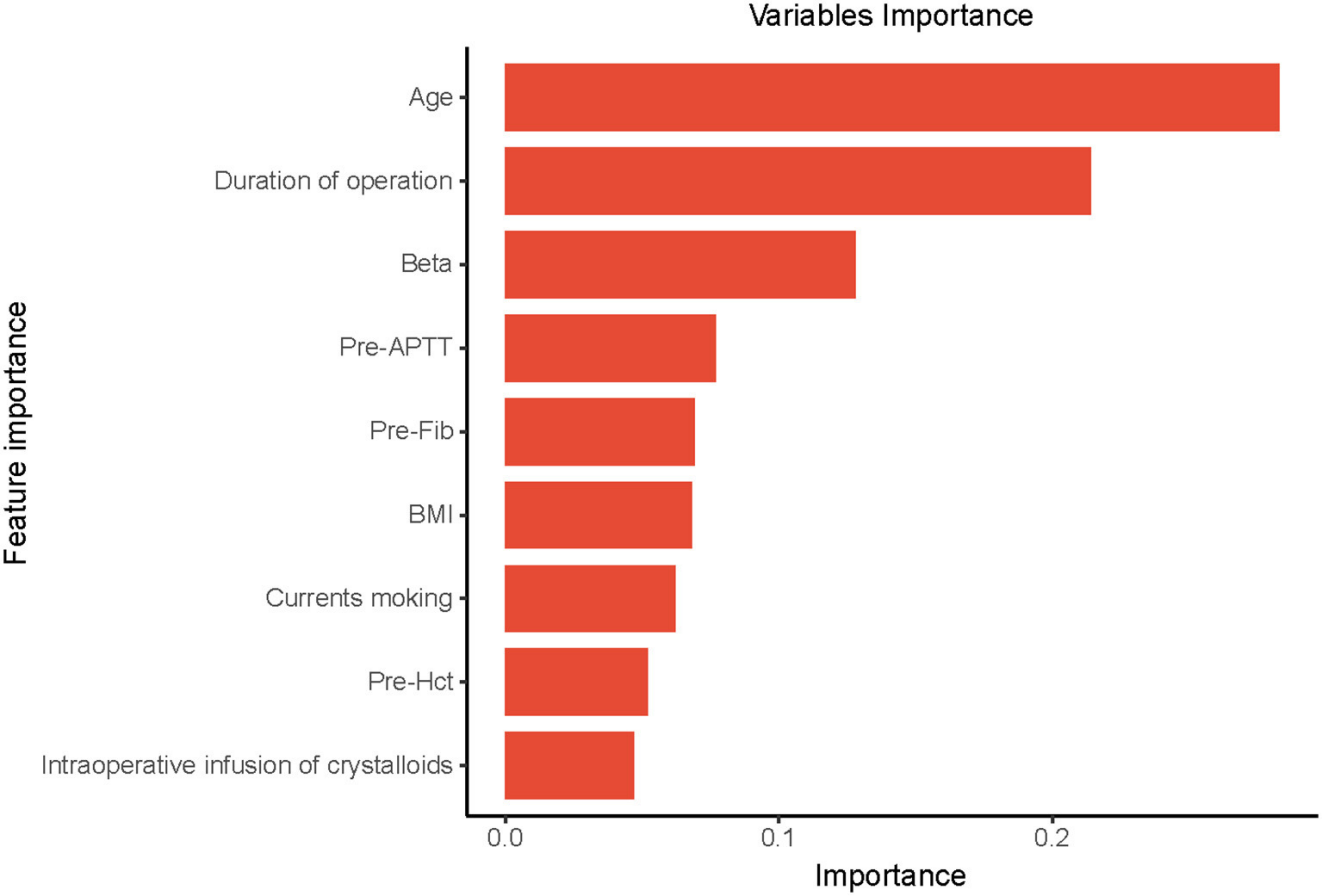
The importance of the variables in the Random Forest (RF) model is in decreasing order as follows: Age, Duration of operation, Beta, Pre-APTT, Pre-Fib, BMI, Current smoking, Pre-Hct, intraoperative infusion of crystalloids. Pre-APTT, pre-operative activated partial thromboplastin time; Pre-Fib, pre-operative fibrinogen; BMI, body mass index; Pre-Hct, pre-operative hematocrit.

Based on the previously mentioned indications, the ultimate prediction model was determined as the RF. Following the RFE, 15 relevant features were obtained in this study, and SHAP values were then utilized to assess the importance. The importance of variables in the RF model showed that age, duration of operation, Beta, pre-operative Fib, and pre-operative APTT were the top five (Figure 5).

## Discussion

In this study, multiple ML-based model algorithms were developed and validated to predict HBL-related risk factors during the TBF perioperative period. We found that the RF model had the best predictive performance compared with other models. In contrast to earlier findings that primarily focused on the risk factors of HBL, our study revealed a highly intriguing finding involved in the importance of risk factors. In this study, the constructed ML model is useful for medical decision-making and clinical management throughout the perioperative period.

HBL refers to undetectable blood loss during surgery, and this part excludes directly quantified blood loss (7). Since the concept of perioperative HBL, problems with HBL-related risk factors have been increasingly noticed by spine surgeons (7). Previous study indicated that consuming a considerable amount of HBL may have serious and adverse consequences (6). Under post-operative stress, increased HBL is followed by a reduction in blood volume, which might excite the sympathetic adrenal medulla system and increase cardiac workload. Furthermore, the kidney may be more vulnerable to ischemia and thus malfunction because of inadequate blood perfusion (21). A substantial quantity of HBL can potentially increase local infection, prolong incision restoration, and delay patient discharge (22). Accordingly, in order to further identify the risk factors for patients' HBL and improve their perioperative management in clinical practice, an increasing amount of research has been designed and carried out, and some of these studies have obtained some meaningful outcomes (5, 6). Using this well-established ML model in this study, we were able to determine the importance of HBL-related risk factors and discovered that the top five risk factors were age, duration of operation, Beta, pre-operative APTT, and pre-operative Fib.

Numerous studies from the past have suggested an association between age and HBL in spine surgery patients (8, 23). Furthermore, age has emerged as the most important risk factor in comparison to other risk factors, as predicted by our ML model. In essence, we are aware that this variable is a risk factor that cannot be modified. With age increasing, bone marrow hematopoiesis and capacity to store red blood cell decline, resulting in poor compensatory ability for blood loss anemia and an increase level of post-operative Hct (24). Additionally, this will further result in a higher HBL calculated by the gross formula. Meanwhile, poor compensatory capacity of the cardiovascular system is followed by the aging process, which reduces the self-regulation ability of the body under surgery-related stresses (8). Increased blood loss and decreased blood return eventually result from this failure to control and correct for blood loss. Additionally, the bleeding is vulnerable to infiltration into extravascular sites due to a decreased change in coagulation activity in older individuals (25).

Our results revealed that patients in the HBL-positive group required more time for surgery than those in the HBL-negative group. It suggested that duration of operation might be an important risk factor for HBL. At present, there are several reports on the relationship between total operational time and perioperative HBL. A retrospective study reported that patients' HBL increased in direct proportion to the length of the surgery (26). Another retrospective cohort analysis discovered a significant correlation between the quantity of HBL and the overall operating time (27). Our findings were essentially in line with those of others. Increased operation time is a highly unfavorable mechanical factor that might extend the overall period of tissue stretching. Then, local ischemia and the release of inflammatory mediators are therefore exacerbated. Eventually, enhanced inflammatory responses can compromise vascular endothelial structure and raise peripheral capillary permeability. Therefore, in order to potentially lessen the detrimental consequences caused by prolonged operation time, clinicians should pay attention to optimizing the operative plan for a shorter duration of operation.

It was reported in the literature that the percentage of fractured vertebral height restoration (Beta) had a beneficial influence on HBL and was recognized as the most influential risk factor among all medical variables (6). Interestingly, our study similarly discovered that the Beta was a crucial promotor of HBL in patients undergoing surgical treatment. Mechanistically, the more restored the fractured vertebral height is, the bigger the local “cavity” might be (6). This process is known as the “empty shell theory”, and it helps explain the current study's result. Meanwhile, when the damaged vertebral height gradually returns, a bigger fracture gap will form surrounding the shattered vertebral walls. This change in the local site may result in easier blood infiltration into interstitial compartments, enhancing the HBL of patients undergoing spinal surgery.

Fib, also referred to as blood coagulation factor I, is a crucial protein that participates in the clotting cascade. In the presence of thrombin, Fib is further activated and transformed into fibrin, which governs platelet adhesion, activation, and coagulation progression (28). Several retrospective studies indicated that pre-operative Fib was a negative risk factor for patients' HBL during spine surgery (8, 29). Similarly, we obtained the same result in our investigation on the relationship between pre-operative Fib and patients' HBL. Low Fib levels may indicate a hypo-coagulable condition of the blood, which may result in blood seeping into interstitial space and an increase in HBL. Furthermore, APTT is another coagulation activity indicator, and its reduction is regarded as activation of the endogenous coagulation pathway. Most of the literature reported that pre-operative APTT was not correlated to patients' HBL during the perioperative period (8, 26). In contrast, some clinical research has identified APTT as a positive risk factor for HBL in TBF patients, and increased APTT can promote an increase in HBL, which could be explained by hemolysis (6). However, our findings suggest that pre-operative APTT content in the HBL-positive group was markedly lower. The process of clotting a cascade is a sequence of enzymatic reactions in the body that are influenced by coagulation factors and pathways. Therefore, we cautiously speculate that lower pre-operative APTT may be due to the concomitant alteration.

Several previous investigations have reported the potential detrimental effects of HBL on patient prognosis, such as post-operative infection, delayed wound healing, cardiovascular disease, renal dysfunction, and so on, and HBL is positively correlated with these poor outcomes (6, 21, 22). Therefore, the study of risk factors for HBL contributes to clinical practice and patients' prognosis. In this study, through RFE feature selection and model prediction, age, operation time, Beta, pre-operative APTT, and Fib were eventually determined as the five most important risk factors for HBL. These variables may be key reasons for a rise in HBL in patients as well as causing post-operative problems. Therefore, clinicians should pay special attention to it. However, due to the potential limitations of the included clinical indicators, other variables affecting HBL cannot be excluded, and a larger sample size and clinical data are required in the future to address this issue. Our study observed post-operative complications and scoring indicators and discovered a significant difference in post-operative superficial incision infection rate between the two groups. The rise in HBL is thought to promote blood accumulation in the third space, leading to a large fluid drainage volume and a long drainage time, which raises the risk of wound infection. However, because of the scarcity of authoritative literature, the single-center retrospective nature of our study, and the small sample size, this result should be generalized with caution. Furthermore, the model constructed in this study was only used to predict the risk factors of HBL and did not involve the prediction and prognostic evaluation indicators of HBL-related complications. In future studies, we will further explore prognostic indicators of HBL, such as length of hospital stay, wound infection, fracture healing, long-term screw-rod fracture, and other adverse events.

This study has several advantages over other studies seeking to the analysis of HBL-related risk factors during the operation of patients with TBF. First, the ML-based model developed and validated in the present study predicted the importance of HBL-related risk factors, which contributes to spine surgeons identifying and managing the HBL-related risk factors during the perioperative period as soon as possible. Second, ML-based model algorithms are reported to outperform traditional linear models in terms of predictive performance. Comfortingly, the constructed prediction model showed great performance for predicting risk factors, which may assist spine surgeons in making decisions and encourage advances in perioperative management.

However, a few limitations should be noted in this study. First, the results had a certain limitation because of the retrospective nature of the present study. Second, there was missing or incorrect data in this study. Third, the constructed model in this study lacked the external validation, particularly in other regions or countries. Finally, all enrolled patients had single-level TBF and did not have multi-level TBF. In the future study, these limitations are expected to be addressed.

## Conclusion

Collectively, we developed and validated ML algorithms for individualized prediction of HBL-related risk factors in the perioperative period of TBF using pre-operative and intraoperative variables. Furthermore, the model could predict the importance of HBL-related risk factors, which might contribute to clinicians in medical decision-making and perioperative management.

## Data availability statement

The data supporting the findings of this study are available from the corresponding author upon reasonable request.

## Ethics statement

The studies involving human participants were reviewed and approved by the Research Ethics Review Board of the Baoji City Hospital of Traditional Chinese Medical (Baoji, China). The Approval Number was No. 2020YTH8H2.

## Author contributions

BY, LG, and XW collected the data, analyzed the data, and drafted the manuscript. JW, BX, and XL supervised the project and reviewed the manuscript. PZ was responsible for the whole project, designed the study, and supervised the study.

## Conflict of interest

The authors declare that the research was conducted in the absence of any commercial or financial relationships that could be construed as a potential conflict of interest.

## Publisher's note

All claims expressed in this article are solely those of the authors and do not necessarily represent those of their affiliated organizations, or those of the publisher, the editors and the reviewers. Any product that may be evaluated in this article, or claim that may be made by its manufacturer, is not guaranteed or endorsed by the publisher.

## Abbreviations

APTT: Activated partial thromboplastin time
ALB: Albumin
AUC: Area under the receiver operating characteristic
BMI: Body mass index
COPD: Chronic obstructive pulmonary disease
CV: Cross-validation
EMR: Electronic medical record
Fib: Fibrinogen
Hb: Hemoglobin
HBL: Hidden blood loss
Hct: Hematocrit
IBL: Intraoperative blood loss
KNN: k-nearest neighbors
ML: Machine learning
MLP: Multi-layer perceptron neural network
NPV: Negative prediction value
PBV: Patient's blood volume
RFE: Recursive feature elimination
PPV: Positive prediction value
SHAP: Shapley additive explanations
SVM: Support vector machine
TBF: Thoracolumbar burst fracture
TBL: Total blood loss
VBL: Visible blood loss.
