# Supplementary material for: Application of supervised machine learning algorithms to predict the risk of hidden blood loss during the perioperative period in thoracolumbar burst fracture patients complicated with neurological compromise
Source: Front Public Health. 2022 Sep 26;10:969919. doi: 10.3389/fpubh.2022.969919 (PMC9549349; doi:10.3389/fpubh.2022.969919)

**Figure S1. The VAS, JOA, and SF-12 scores between the positive-HBL and negative-HBL groups at different time points.** The values are shown as means ± standard deviations (SD). VAS, visual analogue score; JOA, Japanese Orthopedic Association; SF-12, 12-Item Short Form Health Survey; HBL, hidden blood loss.

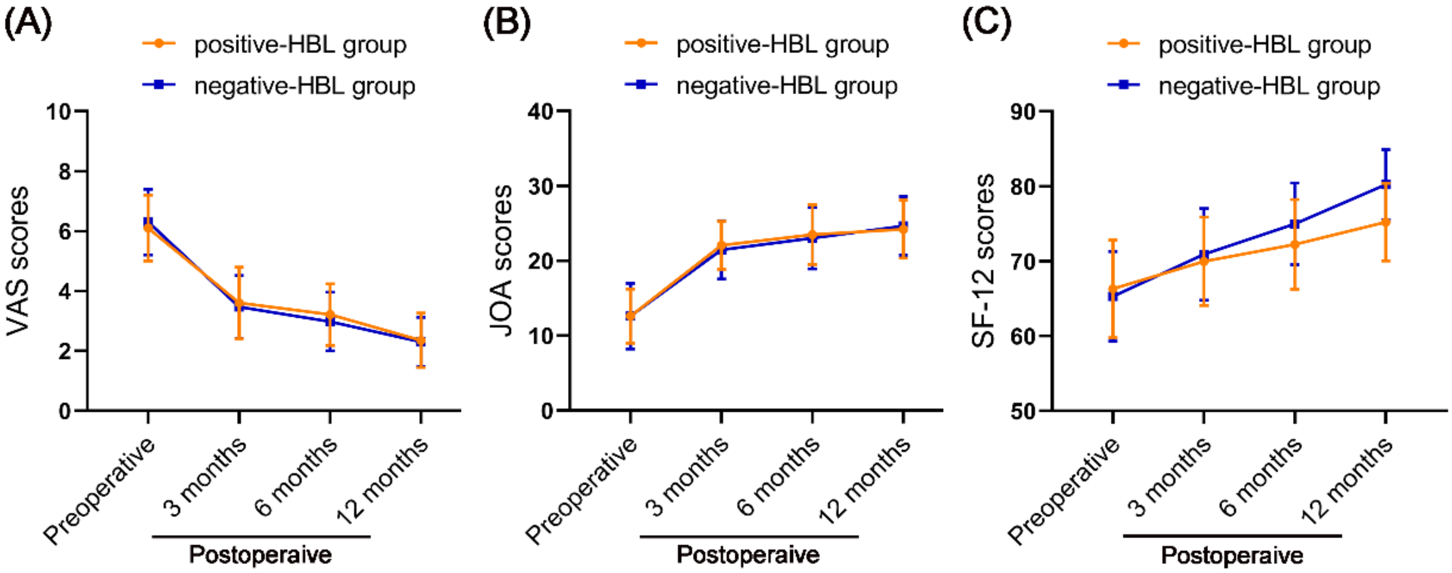

Supplement: Supplementary file 1 [file Table_1.pdf]
